# Extraction and Fractionation of Prokinetic Phytochemicals from *Chrozophora tinctoria* and Their Bioactivities

**DOI:** 10.3390/molecules27134321

**Published:** 2022-07-05

**Authors:** Arshad Iqbal, Ayaz Ali Sher, Naveed Muhammad, Syed Lal Badshah, Abdul-Hamid Emwas, Mariusz Jaremko

**Affiliations:** 1 Department of Botany, Islamia College University, Peshawar 25120, Pakistan; ayazalisher@yahoo.com; 2 Department of Pharmacy, Abdul Wali Khan University, Mardan 23200, Pakistan; drnaveedrph@gmail.com; 3 Department of Chemistry, Islamia College University, Peshawar 25120, Pakistan; 4 Core Laboratories, King Abdullah University of Science and Technology, Thuwal 23955, Saudi Arabia; abdelhamid.emwas@kaust.edu.sa; 5 Smart-Health Initiative (SHI) and Red Sea Research Center (RSRC), Division of Biological and Environmental Sciences and Engineering (BESE), King Abdullah University of Science and Technology (KAUST), Thuwal 23955-6900, Saudi Arabia

**Keywords:** *Chrozophora tinctoria*, acetylcholinesterase inhibitor, laxative, acute toxicity, spasmolytic

## Abstract

*Chrozophora tinctoria* is an annual plant of the family Euphorbiaceae, traditionally used as a laxative, a cathartic and an emetic. A methanolic extract of *Chrozophora tinctoria* (MEC) whole plant and an *n*-butanol fraction of *Chrozophora tinctoria* (NBFC) were analyzed by gas chromatography–mass spectrometry (GC-MS) to detect the phytochemicals. MEC and NBFC were tested for in vitro anti acetylcholinesterase (AChE) potential. The effect of both samples on intestinal propulsive movement and spasmolytic activity in the gastrointestinal tract (GIT) was also studied. About twelve compounds in MEC and three compounds in NBFC were tentatively identified through GC-MS. Some of them are compounds with known therapeutic activity, such as toluene; imipramine; undecane; 14-methyl-pentadecanoic acid methyl ester; and hexadecanoic acid. Both NBFC and MEC samples were checked for acute toxicity and were found to be highly toxic in a dose-dependent manner, causing diarrhea and emesis at 1 g/kg concentration in pigeons, with the highest lethargy and mortality above 3 g/kg. Both the samples of *Chrozophora tinctoria* revealed significant (*p* ≤ 0.01) laxative activity against metronidazole (7 mg/kg) and loperamide hydrochloride (4 mg/kg)-induced constipation. NBFC (81.18 ± 2.5%) and MEC (68.28 ± 2.4%) significantly increased charcoal meal intestinal transit compared to distal water (41.15 ± 4.3%). NBFC exhibited a significant relaxant effect (EC_50_ = 3.40 ± 0.20 mg/mL) in spontaneous rabbit jejunum as compared to MEC (EC_50_ = 4.34 ± 0.68 mg/kg). Similarly, the impact of NBFC on KCl-induced contraction was more significant than that of MEC (EC_50_ values of 7.22 ± 0.06 mg/mL and 7.47 ± 0.57 mg/mL, respectively). The present study scientifically validates the folk use of *Chrozophora tinctoria* in the management of gastrointestinal diseases such as constipation. Further work is needed to isolate the phytochemicals that act as diarrheal agents in *Chrozophora tinctoria*.

## 1. Introduction

Constipation is a gastrointestinal disorder characterized by infrequent and difficult bowel movements, partial evacuation of stools and reduced stool number. Constipation can be defined as “unsatisfactory defecation” [1]. Constipation involves many factors such as low fluid intake, low dietary fiber intake, physical inactivity, medications, irregular bowel habits, history of sexual abuse and neurological and hormonal disorders. It is common in women and people of African origin and is more common in older people than in the young. The symptoms connected with constipation are mild and periodic. They may be chronic and difficult to treat. However, some of the long-term problems associated with constipation are hepatic encephalopathy and inguinal hernia. There are two pathophysiologic mechanisms that can explain constipation. In the first one, slow transit constipation, intestinal peristalsis fails to pass luminal material through the colon, allowing more time for bacterial degradation and the reabsorption of salts and water, thus reducing stool number and weight. The second mechanism is functional outlet obstruction. In this mechanism, stools cannot evacuate fully due to abnormal function of the pelvic floor, the anus or the rectum, and thus one feels a sense of incomplete defecation [2]. Laxatives are used to treat constipation to evacuate the bowel without irritation. Laxatives are classified based on their relative potency. The order of potency is from purgative > cathartic > laxative. Laxatives are further assembled according to their mode of action, as either emollients or lubricants, stool softeners, hydrating agents, stimulants and bulk-forming agents or hydrophilic agents. Bulk-forming or hydrophilic laxatives contain dietary fibers which cause the stool to be bulkier and retain more water as well as forming an emollient gel and making it easier for peristaltic action to move the stool forward. Laxatives add water and bulk to the stool, and similarly, the larger stools help the bowel to contract and pass out the stool. Some bulking laxatives are rice bran, ripe papaya and ripe banana [3]. Plant extracts with promising laxative potential have been reported, diminishing characteristics of constipation by improving intestinal motility, defecation frequency and stool weight. Organic leaf extracts of *Ecklonia cava* exerted a significant laxative effect against loperamide-induced constipation in rats, improving stool recovery and gastrointestinal tract motility [4]. Similarly, extracts from leaves of *Malva sylvestris* have also been reported to alleviate constipation in rats [5]. Through scientific investigations, a significant number of potent chemicals have been reported from plants practiced in folk medicine. The plant *Gallicolumba ferruginea* is a widespread shrub growing at high altitude, 1300–2700 m above sea level, and is used in the management of constipation, intestinal parasite infestation and worm expulsion. Traditional therapeutic plants that are used for the curing of constipation are *Agave americana, Aloe vera, Cassia angustifolia, Cucumis sativus, Coffea arabica, Grewia ferruginea, Linum usitatissimum, Medicago sativa, Plantago ovata (Psyllium), Prunus mallus, Rhamnus frangula* and *Rhamnus purshiana* [6]. Different drugs such as Senna, Gaviscon, Exlax and Correctol are used to overcome the problem of constipation [7,8]. The problem associated with constipation is the regulation of GIT motility, and for this purpose, several prokinetic drugs have been designed. The first amongst these, cisapride, was one of the prokinetic agents but was withdrawn due to its effect of increasing cardiac arrhythmias [9]. Such reports have compelled researchers to explore medicinal plants as prime therapeutic alternatives that have less harmful effects compared to the drugs available today.

**Figure 1:**
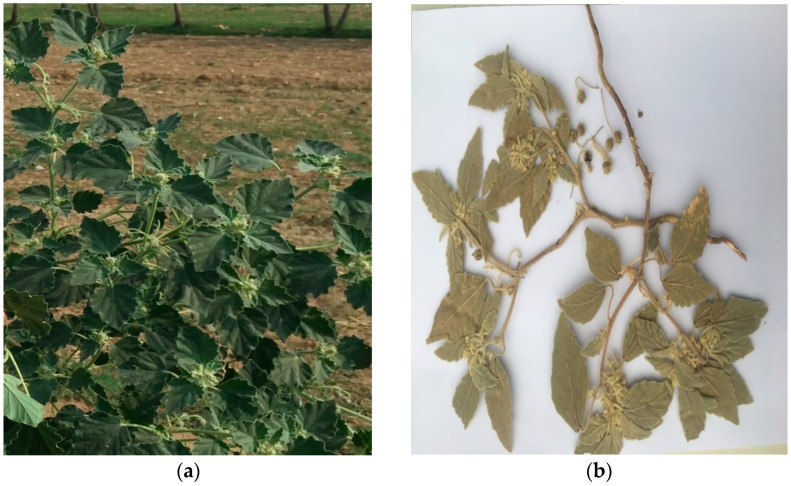
*Chrozophora tinctoria* plant growing in the natural environment (**a**) and dried form (**b**).

*Chrozophora tinctoria* is an annual plant (Figure 1) of the family Euphorbiaceae, sub-family Acalyphoideae, tribe Chrozophoreae and sub-tribe Chrozophorinae. This plant exists in Africa, Europe and tropical parts of Asia including India and Pakistan. The plant produces turnsole, which was used as a coloring agent used in the 15th–17th centuries. It gives luminescence to dyes and pigments. *Chrozophora tinctoria* is used as an antimicrobial, as an antinociceptive, for the treatment of pyrexia and for wound healing in diabetics [10]. Plants of *Chrozophora* are used traditionally as cathartics and emetics, to treat mouth ulcers, warts and skin burns, menstrual problems, GIT worms, abdominal and joint pain, jaundice and migraine. The leaves and seeds of members of the *Chrozophora* are consumed as laxatives in Ethiopia and Senegal, while its fruit juice is taken as a potent remedy for cold in Nepal. The plant is rich in alkaloids, flavonoids, phenylpropanoid glycosides and coumarins. HPLC analysis of the plant confirmed the presence of glucose, fructose, sucrose, raffinose, arabinose and ribose [11]. Almost all the species of *Chrozophora* are rich in flavonoids [12,13,14]. Five flavonoid glycosides have been documented from the aerial portions of *Chrozophora tinctoria* using its methanolic extract. These are: apigenin 7-*O*-β-D-[(6-*p*-coumaroyl)]-glucopyranoside, quercetin 3-*O*-rutinoside, apigenin 7-*O*-β-D-glucopyranoside, apigenin 7-*O*-β-D-[6-(3,4dihydroxybenzoyl)]-glucopyranoside (*Chrozophorine*) and acacetin 7-*O*-rutinoside [15]. These flavonoids possess effective therapeutic potential, including antiviral activity [16,17]. Besides this, about 35 flavonoids have been reported from the genus *Chrozophora,* with profound antioxidant, antipyretic, antimicrobial and antiproliferative activities [10]. In the current study, gas chromatography–mass spectrometry was used to scrutinize the crude methanolic extract and *n*-butanol fractions of *Chrozophora tinctoria* for phytochemicals with laxative activity for the treatment of constipation.

## 2. Results

### 2.1. GC-MS

#### 2.1.1. Composition of MEC

**Figure 2:**
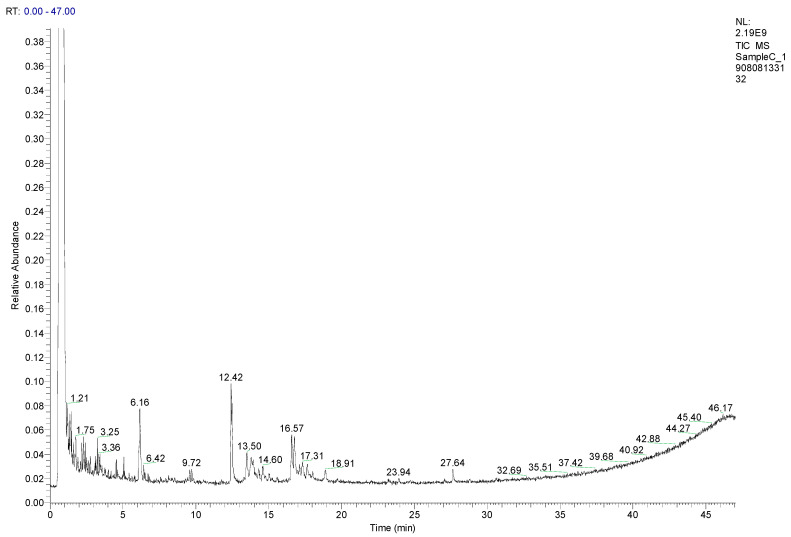
GC-MS chromatogram of methanolic extract of *Chrozophora tinctoria* (MEC). The numbers in the chromatogram are the retention times of the compounds.

About 12 compounds were identified tentatively in crude methanolic extract of *Chrozophora tinctoria* (MEC) as shown in Table 1, Figure 2 and Supplementary Table S1. These compounds include mostly benzene-containing derivatives, long-chain fatty acids, hydrocarbons and the tricyclic antidepressant imipramine. The peaks presented in Figure 1 are in line with the database of phytochemicals present in the GC-MS library. Compounds with a similarity index of more than 600 were selected. Likewise, the GC-MS analysis of MEC manifested the existence of numerous biologically active components at different retention times (minutes).

#### 2.1.2. Composition of NBFC

**Figure 3:**
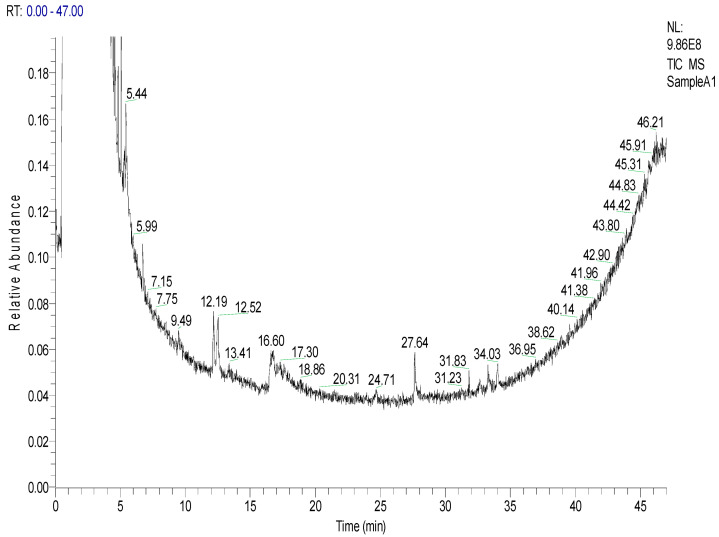
GC-MS chromatogram of the *n*-butanol fraction of *Chrozophora tinctoria* (NBFC). The numbers inside the chromatogram are the retention times of the compounds.

Taking into account the similarity index of more than 600, an NBFC chromatogram (Figure 3) shows the presence of three bioactive compounds, i.e., pidolic acid; pentadecanoic acid, 14-methyl-, methyl ester; and 9-octadecenoic acid, methyl ester, (E), with similarity indexes of 666, 770 and 721 respectively (Table 2). The structures of these three compounds are presented in Supplementary Table S2.

### 2.2. Acetylcholinesterase Inhibitory Activity

#### Effect of MEC and NBFC

MEC and NBFC inhibited AchE at different concentrations as shown in Table 3. At a high concentration of 1000 µg/mL, MEC and NBFC inhibited the enzyme by 83.33 ± 3.51% and 73.33 ± 3.06% respectively. Similarly, the IC_50_ values (µg/mL) measured for MEC and NBFC were 40 and 110, respectively. Galantamine was used as standard with IC_50_ = 5.0 µg/mL.

### 2.3. Acute Toxicity

#### Acute Toxicity of MEC and NBFCT

The acute toxicities of MEC and NBFC are presented in Table 4. Doses of MEC and NBFS up to 0.5 g/kg did not produce toxic symptoms; however, diarrhea and emesis were observed at a concentration of 1 g/kg or more. MEC was emetic and diarrheal at concentrations of 4 and 5 g/kg with 50% mortality, while 50% and 75% mortality were noticed at concentrations of NBFC of 4 g/kg and 5 g/kg.

### 2.4. Laxative Activity

#### 2.4.1. Effect on Metronidazole-Induced Constipation

The laxative (prokinetic) effect of MEC and NBFC was checked against metronidazole-induced constipation. It was observed that metronidazole (Metro) significantly (*p* < 0.001) attenuated the prokinetic effect of MEC by increasing the latency time and reducing the total number of stools, the number of wet stools and the percentage of wet stools, when compared with MEC + DW and control (castor oil) groups as shown in Table 5.

Similarly, the laxative effect (prokinetic effect) of NBFC was also decreased to some extent by metronidazole as shown in Table 6.

#### 2.4.2. Effect of Loperamide-Induced Constipation

MEC and NBFC were tested for laxative effect against loperamide hydrochloride (4 mg/kg)-induced constipation as presented in Table 7 and Table 8, respectively. Loperamide hydrochloride significantly (*p* < 0.001) reduced the prokinetic effect of MEC, either at 1 g/kg or 2 g/kg of MEC. Thus, the percentage of wet stools was significantly lowered either from 52.50 ± 5.63% to 37.51 ± 4.86 *** or from 70.80 ± 1.82% to 55.71 ± 5.15 *** correspondingly. Loperamide hydrochloride exhibited no significant effect against MEC at a concentration of 3 g/kg. A similar pattern of the prokinetic effect was observed for NBFC against loperamide-induced constipation, as shown in Table 8.

### 2.5. Gastrointestinal Motility (Charcoal Meal Method)

To understand the effects of the *Chrozophora tinctoria* fractions on intestinal motility, charcoal meal experiments were performed. The results obtained from the above concentrations were compared with distilled water and castor oil to determine the effect of concentration on intestinal motility. The methanolic extract of *Chrozophora tinctoria* manifested 53.1 ± 5.2% ***, 64.01 ± 5.2% *** and 68.28 ± 2.4% *** of intestine movement while *n*-butanol fraction displayed 45 ± 1.4% *, 66.46 ± 1.5% *** and 81.18 ± 2.5% *** intestinal transit at 25 mg/kg, 50 mg/kg and 100 mg/kg, respectively (Table 9).

### 2.6. Sposmolytic Activity

**Figure 4:**
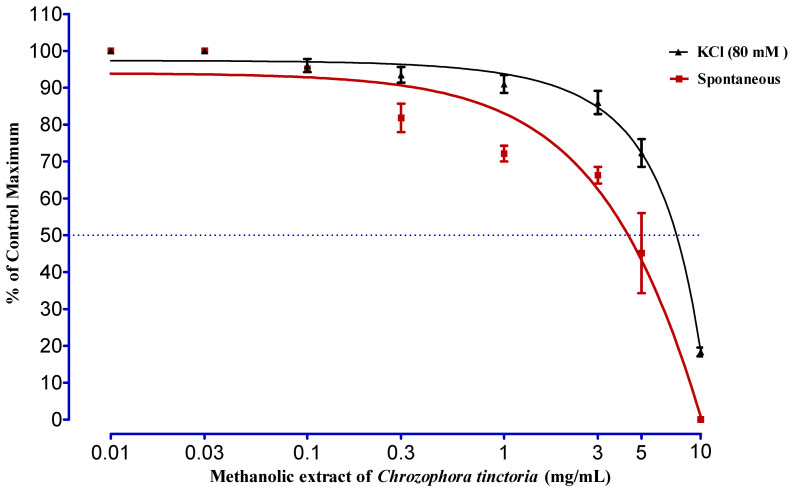
The effect of MEC on spontaneous and KCl-induced (80 mM) contraction of rabbit jejunum. Rabbit jejunum muscle was relaxed in a dose-dependent manner. EC_50_ values were calculated from curve fitting in GraphPad Prism 6.01. Each point represents the mean ± SEM of grouped data.

The methanolic extract of *Chrozophora tinctoria* was tested against spontaneous contraction of rabbit jejunum and KCl-induced (80 mM) contractions (Figure 4). At extract concentrations of 0.01, 0.03, 0.1 and 0.3 mg/mL concentration, no effect was seen. However, from 1 mg/mL to 10 mg/mL, the fraction was very effective in relaxing spontaneous rabbit jejunum contractions. Maximum results were noticed at 5 mg/kg and 10 mg/kg, with an EC_50_ of 4.34 ± 0.68 mg/kg. Similarly, the impact of the same fraction is also observed against a high concentration KCl-induced (80 mM) contraction. It was concluded that the fraction showed a significant relaxing effect at 3, 5 and 10 mg/mL with an EC_50_ of 7.47 ± 0.57 mg/mL.

**Figure 5:**
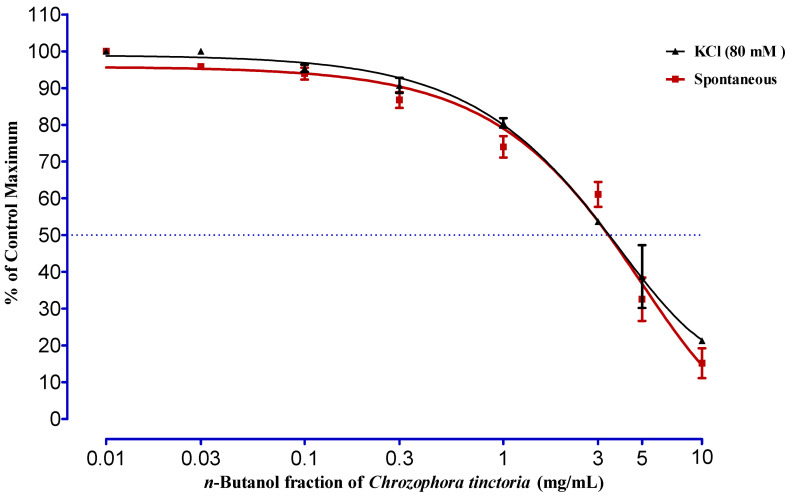
The effect of NBFC on spontaneous and KCl-induced (80 mM) contraction of rabbit jejunum. Rabbit jejunum muscle was relaxed in a dose-dependent manner. EC_50_ values were calculated from curve fitting in GraphPad Prism 6.01. Each point represents the mean ± SEM of grouped data.

As shown in Figure 5, the *n*-butanol fraction of *Chrozophora tinctoria* was checked against spontaneous rabbit jejunum responses. The impression of the fraction started from 0.03 mg/mL to 10 mg/mL, and the maximum relaxing effect was at 10 mg/mL with an EC_50_ value of 3.40 ± 0.20 mg/mL. The fraction with different concentrations was also evaluated against KCl-induced (80 mM) contraction. It was observed that the fraction was less potent at 0.03 mg/mL and was more influential at 10 mg/mL, with an EC_50_ value of 7.22 ± 0.06 mg/mL.

## 3. Discussion

Human beings have used medicinal plants in their traditional systems of medicine for centuries due to their medicinal properties. Natural products collected from microbes have been used as a source of antibiotics, but with the passage of time, there is increasing knowledge about herbal medicine. Herbal medicines have been used for health care, and screening of therapeutic plants for bioactive compounds has become important. Higher plants have been used as a source of active compounds as they are rich sources of secondary metabolites, and some chemotherapeutic agents come from plants. To gain knowledge and analyze medicinal plants to determine their bioactive compounds, GC-MS is normally used [18]. The GC-MS of the methanolic extract and *n*-butanol fraction of *Chrozophora tinctoria* showed the existence of diverse therapeutically active compounds, as shown in Table 1 and Table 2. Bioactive molecules such as toluene; imipramine; undecane; butylated hydroxytoluene; 14-methyl-pentadecanoic acid methyl ester; and hexadecanoic acid methyl ester, were significant and therapeutically active. Toluene has psychoactive effects when inhaled intentionally, and it is also used in paints, plastic production, lacquers, thinners and glues [19]. Imipramine is used to treat depression [20]. Undecane is used as an enzyme inhibitor, carcinogen and antimicrobial, and in various types of cockroaches and moths, it is used as a mild sex attractant [21]. Butylated hydroxytoluene is used as a food preservative and antioxidant [22]. Pentadecanoic acid, 14-methyl-, methyl ester is used as an antifungal, antimicrobial and antioxidant [23]. Hexadecanoic acid methyl ester is used as an antioxidant, nematocide, pesticide, hypocholesterolemic, hemolytic and 5-alpha reductase inhibitor [24].

Constipation is the most common gastrointestinal disorder in which an individual faces exceptional bowel movements with comparatively dry and hard stools occurring less than three times a week. About two million cases of this gastrointestinal complaint are reported in the world annually. Constipation causes include lack of liquids, lack of fiber in the diet, side effects of medications, irritable bowel syndrome, lack of exercise and other factors such as pregnancy and old age [25]. Constipation can be controlled by using nonpharmacologic agents such as sufficient liquid intake and use of dietary fibers and/or pharmacological agents used to control constipation including drugs such as laxatives, opioid antagonists, serotonin receptor agonists and colonic secretagogues [6]. In the current findings, it was found that the crude methanolic extract and importantly *n*-butanol fraction of *Chrozophora tinctoria* reduced constipation. Therefore, they can be used as therapeutic agents against constipation.

Acetylcholine (Ach) is a neurotransmitter in the brain and body organized for cholinergic transmission. Cholinesterase (acetylcholinesterase) belongs to the family of enzymes that hydrolyzes the neurotransmitter acetylcholine into choline and acetic acid. AchE inhibition is an auspicious plan of action against Alzheimer’s disease, myasthenia gravis, Parkinson’s disease, senile dementia and ataxia [26]. The first known acetylcholinesterase inhibitors such as physostigmine, tacrine and donepezil showed moderate development in the cognitive function of Alzheimer’s patients [27]. The bioactive compounds isolated from the plants have also shown inhibitory potential against acetylcholinesterase [28]. MEC and NBFC significantly inhibited AchE at various concentrations. The percent inhibition induced by MEC and NBFC at high concentration (1000 µg/mL) was 83.33 ± 3.51% and 73.33 ± 3.06%, with an inhibition concentration (IC_50_) equal to 40 µg/mL and 110 µg/mL, respectively. Due to the inhibition of AchE, the accumulation of acetylcholine occurs. The availability of acetylcholinesterase in higher concentrations results in a lengthened interaction with muscarinic and nicotinic receptors, keeping up the impulse. The cholinergic response boosts diarrhea through its intracellular signaling passageway [29,30]. The laxative effect of MEC and NBFC was observed in metronidazole (7 mg/kg)- and loperamide hydrochloride (4 mg/kg)-induced constipated pigeons. Both the fractions were found to be diarrheal. NBFC was found more laxative compared to MEC, and this higher prokinetic potential was attenuated by metronidazole more significantly than loperamide hydrochloride. The laxative effect of MEC and NBFC was further validated by the charcoal meal experiment. The effect of both the fractions of *Chrozophora tinctoria* was noted in intestinal motility. It was confirmed that both the fractions exhibited significant (*p* ≤ 0.05) propulsion of charcoal in the pigeon intestine.

The contraction of the intestine is due to intermittent depolarization of smooth muscles due to liberation of Ca^+2^, either from calcium influx or intracellular stores to the inside of the tissue with the help of voltage-gated calcium channels. High-level potassium chloride (KCl) is regarded as a depolarizer that can induce calcium influx into cells. The relaxation produced in KCl-induced contracted muscles generally shows that the tested material influences their activity through calcium channel blockade [31]. This research work was carried out to scrutinize the effect of MEC and NBFC on the smooth muscles of rabbit jejunum. The spasmolytic effect of MEC on the smooth muscles was observed at a concentration of 1 mg/mL. In the same way, the spasm produced by KCl (80 mM) in the jejunum was relaxed significantly at 5 mg/mL and 10 mg/mL with an EC_50_ of 7.47 ± 0.57 mg/mL. Similarly, the effect of NBFC was checked against spontaneous rabbit jejunum responses. The fraction started to have an effect from 0.03 mg/mL to 10 mg/mL, with the maximum relaxing effect at 10 mg/mL with an EC_50_ value of 3.40 ± 0.20 mg/mL. In the case of KCl-induced (80 mM) contraction, it was observed that NBFC was more influential at 10 mg/mL with an EC_50_ value of 7.22 ± 0.06 mg/mL. It was concluded that the relaxing effect of MEC and NBFC against spontaneous and KCl-induced (80 mM) contraction may be due to the inhibition of voltage-gated calcium channels. It proves that NBFC is more potent than MEC. Both the samples were also tested for acute toxicity on pigeons, and it was observed that doses of MEC and NBFC up to 0.5 g/kg were secure and nontoxic. However, MEC with concentrations of 4 and 5 g/kg was found to be emetic and diarrheal with 50% mortality, while 50% and 75% mortality was noticed at 4 g/kg and 5 g/kg concentration NBFC, respectively.

## 4. Materials and Methods

### 4.1. Chemicals and Solvents

The chemicals/drugs used in the present study were Flagyl (Sanofi Aventis (PVT) LTD, Karachi, Pakistan), gum acacia (Shreeji Pharma International, Vadodara, India), castor oil (Karachi Pharmaceuticals Laboratory, Karachi, Pakistan), Immodium (ASPIN Pharma PVT. LTD, Karachi, Pakistan), distilled water, normal saline (Shahzeb Pharmaceutical, Haripur, Pakistan), methanol, *n*-hexane, dichloromethane, ethyl acetate, *n*-butanol (Master chemical supplier, Karachi, Pakistan) and Tyrode solution (Khyber medical university, Peshawar, Pakistan).

### 4.2. Instruments

Instruments used were as follows: chopper machine; electric grinder; large and small flasks; separating funnel; vacuum rotary evaporator (Model RE-111, Labstac LLC, Pittsfield, MA, USA); analytical balance (Shimadzo analytical balance, Karachi, Pakistan); glass funnel; filter papers; disposable syringes of 5 cc, 3 cc, and 1 cc (Shifa disposable syringe), dissection kit (Haq chemicals, Peshawar, Pakistan); drip set (Shifa drip set, Karachi, Pakistan); feeding tube; water bath (Thermostatic controlled-STD/GMP); magnetic stirrer (H3760-S Digital magnetic stirrer); large and small cages; Petri dishes; power lab (Khyber medical university, Peshawar, Pakistan).

### 4.3. Plant Collection and Identification

Full and mature *Chrozophora tinctoria* plants were collected from District Mohmand (34.22° N to 71.48° E), Khyber Pakhtunkhwa, Pakistan, from mid-August to early September 2018. They were authenticated by Sher Wali (PhD), Assistant Prof. Department of Botany, Islamia College Peshawar. Following identification, the plant was given a voucher No. (CT-Bot-11082017), and a specimen was placed in the Herbarium, Department of Botany, Islamia College Peshawar. To clean the collected plant from dirt, it was washed with tap water and dried at room temperature in shade. After complete drying, the plant was cut into pieces and was ground down to powder using an electric grinder.

### 4.4. Extraction and Fractionation

Crude extract preparation: Ten kilograms of the powdered material of the plant was macerated in methanol for a week and agitated three times a day. The mixture was filtered using filter paper. The filtrate was concentrated through a vacuum rotary evaporator at 50 °C. Further drying was performed with a water bath at 50 °C to obtain a crude methanolic extract. The crude methanolic extract was divided into two portions; one was used as a crude methanolic extract, and the other part was fractionated with different solvents. The process of fractionation started by adding distilled water to the crude methanolic extract. After this, *n*-hexane was mixed into the mixture in a separating funnel, shaken gently so that the two layers mixed well and was then allowed to stand for 15 min to separate the two phases. The upper *n*-hexane phase was collected, and the lower aqueous phase in the separating funnel was re-extracted again with fresh *n*-hexane. All the fractions of *n*-hexane were concentrated using a rotary evaporator. A similar procedure was carried out for a series of solvents with increasing polarity, i.e., dichloromethane, ethyl acetate and *n*-butanol, to obtain their fractions. This method is called solvent–solvent fractionation. The crude methanolic fraction obtained in the first phase and *n*-butanol fraction in the last were used for further activities described in this work [32].

### 4.5. Gas Chromatography–Mass Spectrometry (GC-MS)

To understand which phytochemicals are present in the methanolic extract (MEC) and *n*-butanol fraction of *Chrozophora tinctoria* (NBFC), both samples were subjected to gas chromatography–mass spectrometry [33]. The plant samples were checked using a Thermo Scientific (DSQ-II) GC, furnished with a 30 m long TR-5MS capillary column and a 0.25 µm thick film with 0.25 mm of internal diameter. Helium was used as a carrier gas with a flow rate of 1 mL/min. The injection device was run in a split mode at 250 °C. The sample was injected, 1 µL at a time, with an initial oven temperature of 50 °C that was maintained for 2 min followed by gradually elevating the temperature to 150 °C at a rate of 8 °C/min. Ultimately, the temperature was increased to 300 °C at a speed of 15 °C/min and sustained for 5 min [34,35]. The mass spectrometry was performed in full scan mode to obtain information about mass fragments and mass/charge (*m*/*z*) ratio in the range of 50–600.

### 4.6. Pharmacological Activities

#### 4.6.1. Laxative Activity

Healthy pigeons were arranged into eight groups (*n* = 8). The cages were provided with white, plastic bases for examination and stool collection. Groups 1, 2 and 3 were administered distilled water plus 1 g, 2 g and 3 g doses of both the fractions, respectively. Group 4 was given distilled water (6 mL/kg), and group 5 was given castor oil (6 mL/kg PO). Groups 6, 7 and 8 were given doses of 1 g, 2 g and 3 g of the testing fractions. Metronidazole (7 mg/kg) [36] and loperamide hydrochloride (4 mg/kg) [37] were administered to induce constipation in all the groups except groups 1, 2 and 3. Thirty minutes after, all the parameters, i.e., the first stool time/latency time (min), number of stools, number of wet stools and weight of stools (g), were recorded, and the percent effect was calculated as follows [38,39]:

(1) $$Percent\;inhibition=\;\frac{Number\;of\;wet\;stools\;of\;individual}{Total\;number\;of\;stools\;of\;individual\;}\times 100$$

Healthy (with normal stools, non-lethargic, no nasal dropping, no weight loss, no shedding or feather ruffling and usual flying movement with regular feeding) and mature pigeons of either sex weighing 250–350 g were selected. They were provided a dark/light cycle for 12/12 h for 5–7 days and were allowed to drink fresh water and standard food (locally available food; millet + wheat grains).

On the day of the experiment, the pigeons were weighed and examined again for health condition. Pigeons observed as healthy were picked for the experiment, and those found unhealthy were removed from the experiment. The selected animals were caged separately to record data of individual pigeons of all groups [40]. All the pigeons were held gently, and fractions were administered orally.

#### 4.6.2. In Vitro Experiments

##### Acetylcholinesterase Inhibitory Assay

Antiacetylcholinesterase activity was determined by applying the method of Ellman [41], with increasing concentrations of MEC and NBFC (125, 250, 500, 1000 µg/mL). The basic principle of this protocol is the hydrolysis of acetylthiocholine iodide into thiocholine using the respective enzyme, which reacts with 5, 5′-dithiobis-2-nitrobenzoic acid (DTNB) (Ellman’s reagent). The final products of the second reaction are 5-thio-2 nitrobenzoate and 2-nitrobenzoate-5-mercaptothiocholine. The absorption of the former product was measured with a spectrophotometer (412 nm). Galantamine, the positive control, was applied in the same concentrations as MEC and NBFC. All the tested samples were incubated at 37 °C for 20 min. The enzyme inhibition was calculated from the absorption rate with a change in time [41,42].

The percent enzyme inhibition was calculated as

Enzyme inhibition (%) = 100 − percent enzyme activity(2)

Percent enzyme activity (%) = 100 × V/V_max_ (3)

where (V_max_) is an enzyme activity in the absence of an inhibitor.

#### 4.6.3. In Vivo Experiments

##### Acute Toxicity

To check and establish the toxic effect of MEC and NBFC, we used a previously described protocol with some modifications [43]. The experimental animals (pigeons) were divided into two groups (*n* = 8). One group received plant extracts while the negative control group received distilled water only (6 mL/kg, PO). MEC and NBFC were administered orally with 0.3, 0.5, 1, 2, 3, 4 and 5 g/kg as a single dose to different groups using a feeding tube. All the pigeons were observed for toxic symptoms, i.e., diarrhea, emesis, lethargy and motility, for about 72 h [43].

##### Charcoal Meal Treatment

Pigeons were divided into five groups (*n* = 8). All the groups were constipated by administering loperamide hydrochloride at a dose of 4 mg/kg. Group 1 was given distilled water (2 mL); group 2 was served with castor oil; and groups 3, 4 and 5 were treated with fractions in concentrations of 25, 50, and 100 mg/kg, respectively. After 30 min, 2 mL of charcoal meal (a solution of 10% charcoal and 5% gum acacia) was given orally to each pigeon. All the pigeons were then provided with water and food and were sacrificed after 30 min. After that, the whole intestine, starting from the pylorus region to the ileocecal junction, was removed from the pigeons and placed on white paper parallel to a ruler. The distance traveled by the charcoal marker was measured and expressed as percent intestinal transit [44]. The percent effect was calculated as follows:

(4) $$Percent\;intestinal\;transit=\frac{Distance\;travel\;by\;charcoal}{\;The\;total\;length\;of\;intestine}\times 100$$

##### Spasmolytic Activity

The spasmolytic effect of MEC and NBFC was evaluated according to earlier reported studies [45]. Mature and healthy male rabbits weighing 1.2–3.5 kg were subjected to spasmolytic activity. Briefly, following cervical dislocation [46], the abdomen of the rabbit was opened and the jejunum was cut into slices of 1.5–2.5 cm and placed in Petri plates having Tyrode solution with a continuous supply of carbogen gas (95% O_2_ and 5% CO_2_). The mesentery was removed from the isolated jejunum tissue and was fixed in the organ bath containing Tyrode solution at a retained temperature of 37 ± 1 °C and stabilized for about 20–30 min. The stable tissue with spontaneous response was taken as a baseline control, which is a positive control. The relaxing effect of the tested plant extract was compared. The effect of the fractions on spontaneous activity of the jejunum preparation was observed using different concentrations, i.e., 0.01, 0.03, 0.1, 0.3, 3.0, 5.0 and 10 mg/mL, at an interval of 1 to 2 min in a cumulative manner. The effect of the fractions was also tested against KCl-induced contraction [45].

### 4.7. Ethical Approval

The study was approved by the ethical committee of the Department of Pharmacy, Abdul Wali Khan University, Mardan, Pakistan. The ethical approval No. is EC/PhM/AWKUM-871D.

### 4.8. Statistical Analysis

Data are expressed as the mean. One-way ANOVA followed by Dunnett’s test was applied. The concentration–response curve was plotted using GraphPad Prism for Windows 6.0 (GraphPad Software, San Diego, CA, USA).

### 4.9. List of Abbreviations

MEC: methanolic extract of Chrozophora; NBFC: *n*-butanol fraction of Chrozophora; KCl: potassium chloride; AchE: acetylcholine esterase; KPK: Khyber Pakhtunkhwa; DTNB: 5,5′-dithiobis-2-nitrobenzoic acid; PO: per orally; DW: distilled water; HPLC: high-performance liquid chromatography; GC-MS: gas chromatography–mass spectrometry.

## 5. Conclusions

Processed and refined food and low intake of fiber cause constipation and other related problems such as hemorrhoids. The utilization of herbal products with low side effects is always beneficial for the improvement of health. The different parts of herbs possess special phytochemicals that have unique biological activity. Chrozophora plants contain several important bioactive molecules which have laxative and antidepressant properties. Utilization of this plant in tea and other drinks can be useful. However, overutilization may have other side effects that should be avoided. Further work is needed to isolate the phytochemicals that act as diarrheal agents in *Chrozophora tinctoria.*

## Figures and Tables

**Table 1:** Bioactive compounds identified in methanolic extract of *Chrozophora tinctoria* by GC-MS.

Peak No.	Retention Time (min)	SI	RSI	Area %	Probability	Compound Name	Formula	Molecular Weight	Library
1	1.21	828	893	0.23	25.72	Toluene	C_7_H_8_	92	Replib
2	1.75	943	943	19.37	41.03	o-Xylene	C_8_H_10_	106	Replib
3	3.25	641	976	0.02	36.57	Imipramine	C_19_H_24_N_2_	280	nist_msms
4	3.36	606	802	0.15	23.31	Undecane	C_11_H_24_	156	Replib
5	6.16	787	851	0.04	48.08	Butylated Hydroxytoluene	C_15_H_24_O	220	Replib
6	6.42	834	899	0.18	58.97	Pentadecanoic acid, 14-methyl-, methyl ester	C_17_H_34_O_2_	270	MAINLIB
7	9.72	629	652	0.27	52.21	Nonanoic acid, 9-(o-propylphenyl)-, methyl ester	C_19_H_30_O_2_	290	MAINLIB
8	12.42	897	908	0.45	73.48	Hexadecanoic acid, methyl ester	C_17_H_34_O_2_	270	Replib
9	16.57	799	812	0.55	8.15	10-Octadecenoic acid, methyl ester	C_19_H_36_O_2_	296	MAINLIB
10	17.31	630	724	0.04	25.25	Heptadecanoic acid, 9-methyl-, methyl ester	C_19_H_38_O_2_	298	MAINLIB
11	18.91	635	667	0.01	30.39	Spirost-8-e*n*-11-one, 3-hydroxy-, (3á,5à,14á,20á,22á,25R)-	C_27_H_40_O_4_	428	MAINLIB
12	27.64	674	718	0.01	41.20	1-Monolinoleoylglycerol trimethylsilyl ether	C_27_H_54_O_4_Si_2_	498	MAINLIB

SI: similarity index; RSI: relative similarity index.

**Table 2:** Bioactive compounds identified in the *n*-butanol fraction of *Chrozophora tinctoria* through GC-MS.

Peak No.	Retention Time (min)	SI	RSI	Area %	Probability	Compound Name	Formula	Class	Molecular Weight	Library
1	5.44	666	885	0.01	12.91	Pidolic Acid	C_5_H_7_NO_3_	Amino acid derivative	129	MAINLIB
2	12.19	770	867	0.01	67.68	Pentadecanoic acid, 14-methyl-, methyl ester	C_17_H_34_O_2_	Fatty acid	270	MAINLIB
3	16.60	721	788	0.01	8.72	9-Octadecenoic acid, methyl ester, (E)-	C_19_H_36_O_2_	Fatty acid	296	Replib

**Table 3:** Percent inhibition of acetylcholinesterase by MEC and NBFC.

Compound/Plant	Extract/Fraction	Concentration (µg/mL)	% AChE Inhibition	IC_50_ (µg/mL)
Galantamine	Standard	1000	95.67 ± 2.52	5.0
		500	87.33 ± 2.52	
		250	82.67 ± 3.06	
		125	77.00 ± 3.00	
*Chrozophora* *tinctoria*	MEC	1000	83.33 ± 3.51	40
		500	73.00 ± 3.00	
		250	68.33 ± 4.51	
		125	61.00 ± 2.00	
	NBFC	1000	73.33 ± 3.06	110
		500	65.00 ± 2.65	
		250	57.67 ± 2.08	
		125	50.33 ± 2.52	

Values are expressed as mean ± SEM. Statistical significance was determined using IC_50_ values through Biostata software. IC_50_ = half-maximal inhibitory concentration; MEC= methanolic extract of *C. tinctoria;* AChE = Acetylcholinesterase; NBFC = *n*-butanol fraction of *Chrozophora tinctoria*.

**Table 4:** Acute toxicity of methanolic extract and *n*-butanol fractions of *Chrozophora tinctoria* in pigeons.

Sample	Dose (g)·(mL)/kg	Emesis	Diarrhea	Lethargy	Mortality (%)
		Total Number of Vomits	Total Number of Wet Stools		
Distal Water	6	0.00 ± 0.00	0.00 ± 0.00	-	0.00 ± 0.00
MEC	0.3	0.00 ± 0.00	0.00 ± 0.00	-	0.00 ± 0.00
	0.5	0.00 ± 0.00	0.00 ± 0.00	-	0.00 ± 0.00
	1	4.00 ± 1.00	9.33 ± 2.52 *	-	0.00 ± 0.00
	2	5.00 ± 2.00	13.33 ± 2.52 ***	-	0.00 ± 0.00
	3	7.00 ± 2.00 **	14.00 ± 4.58 ***	Less	0.00 ± 0.00
	4	7.67 ± 2.52 **	14.33 ± 4.51 ***	More	50.00 ± 0.00
	5	9.00 ± 2.65 ***	16.33 ± 2.52 ***	More	50.00 ± 0.00
NBFC	0.3	0.00 ± 0.00	0.00 ± 0.00	-	0.00 ± 0.00
	0.5	0.00 ± 0.00	0.00 ± 0.00	-	0.00 ± 0.00
	1	3.00 ± 1.00	10.33 ± 3.51 *	-	0.00 ± 0.00
	2	4.67 ± 1.53	14.67 ± 3.06 ***	Less	0.00 ± 0.00
	3	5.00 ± 1.73	15.00 ± 5.00 ***	More	25.00 ± 0.00
	4	6.00 ± 2.00 *	16.00 ± 3.61 ***	Most	50.00 ± 0.00
	5	7.33 ± 3.51 **	19.33 ± 3.51 ***	Most	75.00 ± 0.00

Values are expressed as mean ± SEM. Statistical significance was determined using GraphPad Prism 6.01 software (one-way ANOVA followed by Tukey’s multiple comparison test), by Dotmatics, San Diego, CA, USA. * *p* ≤ 0.05 was considered statistically significant. (** *p* ≤ 0.01, *** *p* ≤ 0.001). MEC = methanolic extract of *C. tinctoria*; AChE = Acetylcholinesterase; NBFC = *n*-butanol fraction of *Chrozophora tinctoria*.

**Table 5:** Diarrheal/laxative activity of methanolic extract of *C. tinctoria* in metronidazole induced consti-pation in pigeons.

Samples	Dose g/kg ML/kg (P/O)	First Stool/Latency Time (Minutes)	Total Number of Stools	Number of Wet Stools	Weight of Stools (Grams)	Percent of Wet Stools (%)
MEC + DW	1	28.67 ± 2.08	17.00 ± 2.00	9.00 ± 2.00	13.27 ± 1.72	52.50 ± 5.63
	2	27.33 ± 2.52	18.33 ± 2.52	13.00 ± 2.00	14.23 ± 2.06	70.80 ± 1.82
	3	25.00 ± 2.00	20.33 ± 0.58	15.00 ± 3.00	17.37 ± 2.07	73.57 ± 12.91
Metronidazole (7 mg/kg) was administered (P.0) 30 min before extract/distal water/castor oil						
DW (-ive Control)	6	73.33 ± 2.52	9.33 ± 2.52	00 ± 00	8.27 ± 1.96	00 ± 00
Castor oil (+ive Control)	6	20.33 ± 2.52 ***	22.00 ± 2.00 ***	18.33 ± 1.53 ***	18.67 ± 2.36 ***	83.43 ± 2.23 ***
MEC + Metro	1	31.33 ± 1.53 ***	14.00 ± 1.00 *	6.33 ± 1.53 ***	10.60 ± 1.75	44.88 ± 7.64 ***
	2	35.33 ± 2.52 ***	15.67 ± 1.53 **	8.67 ± 1.53 ***	12.33 ± 1.22 *	55.02 ± 4.54 ***
	3	37.33 ± 2.52 ***	15.33 ± 1.53	9.33 ± 1.53 ***	12.27 ± 1.32	60.86 ± 3.36 ***

Data are presented as mean ± SEM; one-way ANOVA was done followed by Dunnett’s test to determine statistical significance where *p* ≤ 0.05 was considered statistically significant (* *p* ≤ 0.05; ** *p* ≤ 0.01; *** *p* ≤ 0.001). MEC, methanolic extract of *Chrozophora tinctoria*; Metro, metronidazole; DW, distilled water; PO, per orally/by orally. Two asterisk (**) means data is significant compared to control while three asterisk (***) means its data is more significant compared to control.

**Table 6:** Diarrheal/laxative activity of n-butanol fraction *C. tinctoria* in metronidazole induced constipa-tion in pigeons.

Samples	Dose g/kg mL/kg (P/O)	First Stool/Latency Time (Minutes)	Total Number of Stools	Number of Wet Stools	Weight of Stools (Grams)	Percent of Wet Stool (%)
NBFC + DW	1	28.33 ± 2.52	17.00 ± 3.00	10.33 ± 1.53	13.37 ± 1.96	61.03 ± 2.87
	2	26.33 ± 3.51	19.00 ± 2.00	14.33 ± 1.53	16.13 ± 2.70	75.45 ± 1.54
	3	23.33 ± 2.52	20.00 ± 1.00	16.67 ± 1.15	17.17 ± 2.16	83.31 ± 2.96
Metronidazole (7 mg/kg) was administered (P.0) 30 min before fractions/distilled water/castor oil						
DW (−ive Control)	6	73.33 ± 2.52	9.33 ± 2.52	00 ± 00	8.27 ± 1.96	00 ± 00
Castor oil (+ive Control)	6	20.33 ± 2.52 ***	22.00 ± 2.00 ***	18.33 ± 1.53 ***	18.67 ± 2.36 ***	83.43 ± 2.23 ***
NBFC + METRO	1	29.33 ± 1.53 ***	15.33 ± 0.58 **	7.67 ± 0.58 ***	12.37 ± 1.56 *	50.00 ± 3.34 ***
	2	30.67 ± 2.08 ***	17.00 ± 2.65 ***	11.67 ± 1.53 ***	15.53 ± 1.60 ***	64.72 ± 2.09 ***
	3	21.67 ± 2.52 ***	17.00 ± 2.00 ***	13.67 ± 1.53 ***	16.10 ± 2.05 ***	80.43 ± 1.75 ***

Data are presented as mean ± SEM; one-way ANOVA followed by Dunnett’s test was done to determine statistical significance where *p* ≤ 0.05 was considered statistically significant (* *p* ≤ 0.05; ** *p* ≤ 0.01; *** *p* ≤ 0.001). NBFC, *n*-butanol fraction of *Chrozophora tinctoria*; Metro, metronidazole; DW, distilled water; PO, per orally/by orally.

**Table 7:** Diarrheal/laxative activity of methanolic extract of *C. tinctoria* in loperamide hydrochloride in-duced constipation in pigeons.

Samples	Dose g/kg mL/kg (P/O)	First Stool/Latency Time (Minutes)	Total Number of Stools	Number of Wet Stools	Weight of Stools (Grams)	Percent of Wet Stool (%)
MEC + DW	1	28.67 ± 2.08	17.00 ± 2.00	9.00 ± 2.00	13.27 ± 1.72	52.50 ± 5.63
	2	27.33 ± 2.52	18.33 ± 2.52	13.00 ± 2.00	14.23 ± 2.06	70.80 ± 1.82
	3	25.00 ± 2.00	20.33 ± 0.58	15.00 ± 3.00	17.37 ± 2.07	73.57 ± 12.91
Loperamide hydrochloride (4 mg/kg) was administered (P.0) 30 min before fractions/distilled water/castor oil						
DW (-ive Control)	6	70.33 ± 2.52	8.33 ± 2.52	00 ± 00	7.50 ± 2.29	00 ± 00
Castor oil (+ive Control)	6	17.33 ± 2.52 ***	16.00 ± 2.00 **	14.33 ± 1.53 ***	15.07 ± 1.27 ***	89.74 ± 2.78 ***
MEC + Lopr	1	38.00 ± 3.00 ***	11.33 ± 2.52	4.33 ± 1.53 ***	8.90 ± 1.65	37.51 ± 4.86 ***
	2	25.00 ± 2.52 ***	13.67 ± 1.53 *	7.67 ± 1.53 ***	11.00 ± 0.95	55.71 ± 5.15 ***
	3	28.33 ± 2.52 ***	13.67 ± 1.53 *	10.00 ± 1.00 ***	12.77 ± 1.37 **	73.15 ± 2.44 ***

Data are represented as mean ± SEM. The data were analyzed by one-way ANOVA followed by Dunnett’s test using GraphPad Prism version 6.01; *p* ≤ 0.05 was considered significant (* *p* ≤ 0.05; ** *p* ≤ 0.01; *** *p* ≤ 0.001). MEC, methanolic extract of *Chrozophora tinctoria*; Lopr, loperamide hydrochloride; DW, distilled water; PO, per orally/by orally.

**Table 8:** Diarrheal/laxative activity of n-butanol fraction of C. tinctoria in loperamide hydrochloride in-duced constipation in pigeons.

Samples	Dose g/kg mL/kg (P/O)	First Stool/Latency Time (Minutes)	Total Number of Stools	Number of Wet Stools	Weight of Stools (Grams)	Percent of Wet Stool (%)
NBFC + DW	1	28.33 ± 2.52	17.00 ± 3.00	10.33 ± 1.53	13.37 ± 1.96	61.03 ± 2.87
	2	26.33 ± 3.51	19.00 ± 2.00	14.33 ± 1.53	16.13 ± 2.70	75.45 ± 1.54
	3	23.33 ± 2.52	20.00 ± 1.00	16.33 ± 1.53	17.17 ± 2.16	81.55 ± 3.64
Loperamide hydrochloride (4 mg/kg) was administered (P.0) 30 min before fractions/distilled water/castor oil						
Distilled Water (-ive Control)	6	70.33 ± 2.52	8.33 ± 2.52	00 ± 00	7.50 ± 2.29	00 ± 00
Castor oil (+ive Control)	6	17.33 ± 2.52 ***	16.00 ± 2.00 **	14.33 ± 1.53 ***	15.07 ± 1.27 ***	89.74 ± 2.78 ***
NBFC + Lopr	1	32.33 ± 2.52 ***	12.67 ± 2.08	5.67 ± 1.53 **	9.50 ± 1.65	44.34 ± 7.11 ***
	2	30.67 ± 2.08 ***	16.33 ± 2.52 ***	8.33 ± 1.53 ***	11.33 ± 1.15 *	50.88 ± 1.52 ***
	3	24.33 ± 2.52 ***	15.00 ± 2.00 **	13.33 ± 1.53 ***	14.00 ± 2.17 ***	89.06 ± 2.91 ***

Data are represented as mean ± SEM. The data were analyzed by one-way ANOVA followed by Dunnett’s test using GraphPad Prism version 6.01; *p* ≤ 0.05 was considered significant (* *p* ≤ 0.05; ** *p* ≤ 0.01; *** *p* ≤ 0.001). NBFC, *n*-butanol fraction of *Chrozophora tinctoria*; Lopr, loperamide hydrochloride; DW, distilled water; PO, per orally/by orally.

**Table 9:** Percent intestinal transit of MEC and NBFC in pigeons.

Treatment	Dose (mg)·(mL)/Kg (PO)	Total Length of Intestine (cm)	Total Distance Travelled by Charcoal Meal (cm)	% Intestinal Transit
Castor oil	6	88.9 ± 5.1	77.7 ± 5.1	87.60 ± 5.8 ***
Distilled Water	6	90.9 ± 2.7	37.3 ± 2.8	41.15 ± 4.3
MEC	25	76.4 ± 2.7	40.5 ± 3.0	53.1 ± 5.2 ***
	50	71.4 ± 2.4	45.6 ± 2.2	64.01 ± 5.2 ***
	100	81.46 ± 1.8	55.6 ± 2.3	68.28 ± 2.4 ***
NBFC	25	88.3 ± 2.01	45.2 ± 1.9	45 ± 1.4 *
	50	83.3 ± 2.9	55.4 ± 2	66.46 ± 1.5 ***
	100	81.4 ± 2.4	66.1 ± 2	81.18 ± 2.5 ***

Data are represented as mean ± SEM (* *p* ≤ 0.05; *** *p* ≤ 0.001). MEC, methanolic extract of *Chrozophora tinctoria*; NBFC, *n*-butanol fraction of *Chrozophora tinctoria*; DW, distilled water; PO, per orally/by orally; %, percentage.

## Data Availability

Data will be provided upon request.

## Supplementary Materials

The following supporting information can be downloaded at: https://www.mdpi.com/article/10.3390/molecules27134321/s1, Table S1: Structures of compounds of methanolic extract of *Chrozophora tinctoria* (MEC); Table S2: Structures of compounds of n-Butanol extract of *Chrozophora tinctoria* (NBFC).

## Sample Availability

Samples of the extracts are available from the authors.
