# Supplementary material for: Extraction and Fractionation of Prokinetic Phytochemicals from Chrozophora tinctoria and Their Bioactivities
Source: Molecules. 2022 Jul 5;27(13):4321. doi: 10.3390/molecules27134321 (PMC9268473; doi:10.3390/molecules27134321)
Supplement: Supplementary file 1 [file molecules-27-04321-s001.zip › molecules-1754713-supplementary.pdf]

Table S1. Structures of compounds of methanolic extract of *Chrozophora tinctoria* (MEC).

S.No	Compound	Group	Structure
1	Toluene	Benzene Derivative	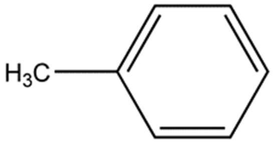
2	o-Xylene	Benzene Derivative	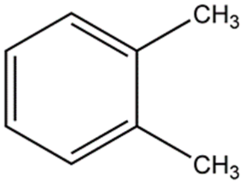
3	Imipramine	Benzene Derivative	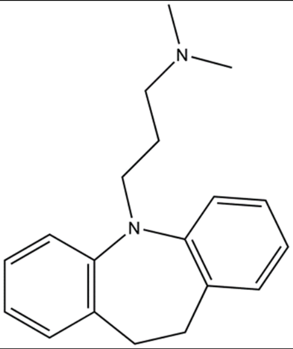
4	Undecane	Hydrocarbon	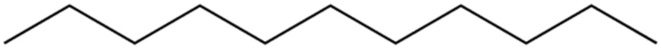
5	Butylated Hydroxytoluene	Benzene Derivative	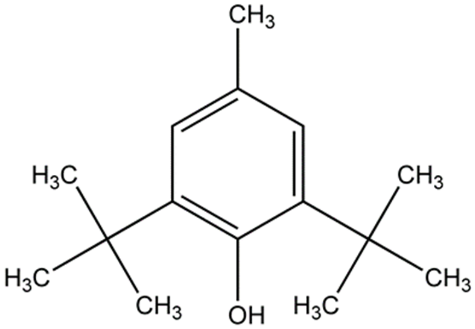
6	Pentadecanoic acid, 14-methyl-, methyl ester	Fatty Acid	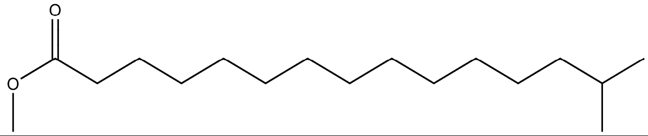
7	Nonanoic acid, 9-(o-propylphenyl)-, methyl ester	Fatty Acid and Benzene derivative congugate	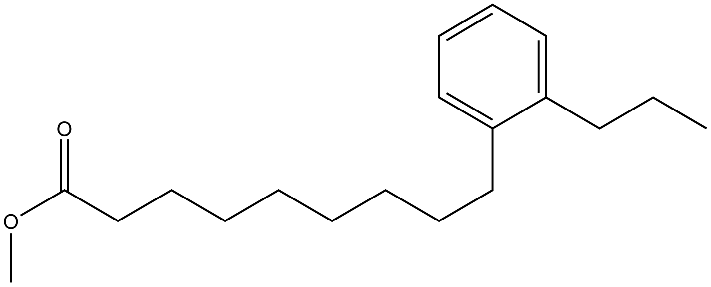

8	Hexadecanoic acid, methyl ester	Fatty Acid	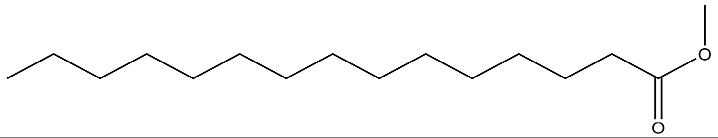
9	10-Octadecenoic acid, methyl ester	Fatty Acid	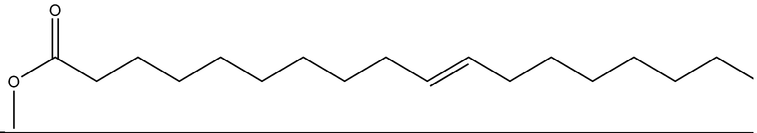
10	Heptadecanoic acid, 9-methyl-,methyl ester	Fatty Acid	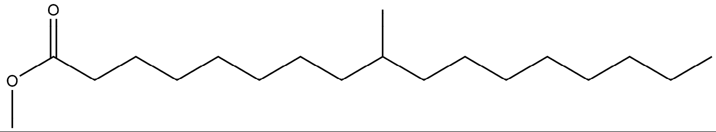
11	Spirost-8-e*n*-11-one, 3-hydroxy-, (3á,5à,14á,20á,22á,25R)-	Hydrocarbon derivative	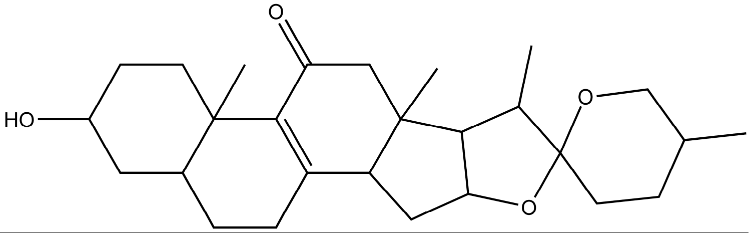
12	1-Monolinoleoylglycerol trimethylsilyl ether	Complex Fatty Acid	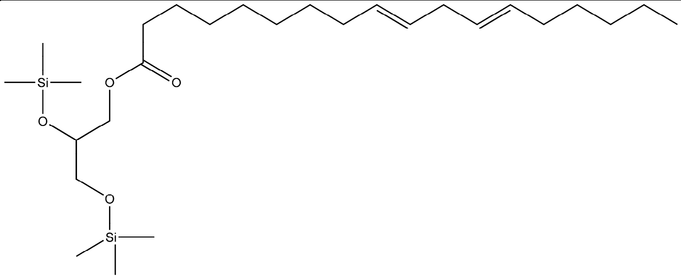

Table S2. Structures of compounds of n-Butanol extract of *Chrozophora tinctoria* (NBFC).

S.No	Compound name	Structure
1	Pidolic Acid	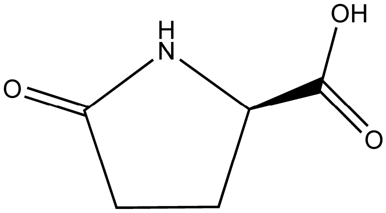
2	Pentadecanoic acid, 14-methyl-, methyl ester	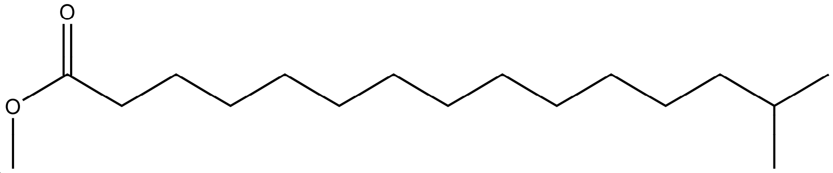
3	9-Octadecenoic acid, methyl ester, (E)-	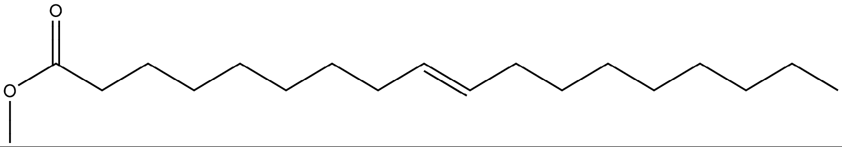
